# Editorial for the Special Issue, “Chemistry of Essential Oils and Food Flavours”

**DOI:** 10.3390/foods11152182

**Published:** 2022-07-22

**Authors:** Ian Southwell, Oscar Núñez

**Affiliations:** 1 Southern Cross Plant Science, Faculty of Science and Engineering, Southern Cross University, Lismore, NSW 2480, Australia; 2 Department of Chemical Engineering and Analytical Chemistry, Faculty of Chemistry, University of Barcelona, Martí i Franquès 1-11, E-08028 Barcelona, Spain; oscar.nunez@ub.edu; 3 Research Institute in Food Nutrition and Food Safety, University of Barcelona, Recinte Torribera, Av. Prat de la Riba 171, Edifici de Recerca (Gaudí), Santa Coloma de Gramenet, E-08921 Barcelona, Spain

Essential oils have important functions in nature. In addition, they are used commercially in categories such as flavors, fragrances, and health-care products. Their properties present a challenge when investigating their chemical composition, function, bioactivity, analysis, and commercialization as value-added products. Analytical techniques for the investigation of essential oil chemistry are continuously evolving. The sophistication, adulteration, or imitation of natural products is becoming difficult to detect as the perpetrators employ modern techniques. Consequently, an understanding of the chemistry of essential oils that keeps abreast with current instrumental and computational developments is paramount.

Thus, the objective of this Special Issue is to broadcast some of the latest progress in essential oil discoveries concerning oil chemistry, methodology, instrumentation, bioactivity, chemical ecology, biosynthesis, and authentication, especially regarding foods.

This Special Issue will be useful for all readers in terms of the novel information it provides on the chemistry of essential oils and food flavors. 

The special issue includes five Research Articles and two Reviews, which are briefly described as follows:

(1) *Rosmarinus officinalis* L. (also known as *Salvia rosmarinus* Schleid) and *Lavandula angustifolia* Mill. belong to the Lamiaceae family, which comprises different genera whose biological activities are used in traditional medicines worldwide. *R. officinalis* has many different uses; its volatile essential oil (EO) and leaf extracts possess extensively studied biological properties, such as antioxidant, anti-inflammatory, antiproliferative, anticancer, and antiviral, among others. *R. officinalis* was investigated for its curative properties against ailments caused by biochemical, chemical, or biological agents. Garzoli et al. [1], in the study “Headspace/GC–MS Analysis and Investigation of Antibacterial, Antioxidant and Cytotoxic Activity of Essential Oils and Hydrolates from *Rosmarinus officinalis* L. and *Lavandula angustifolia* Miller”, described the chemical composition and biological activities of these essential oils and their hydrolates. They are rich in bioactive molecules and have potential applications in various fields, including foods and beverages. Although the oils of these species have been thoroughly examined over the years, less is known about their headspace/GC–MS analysis, especially regarding hydrolates.
(2) Furocoumarins (FCs) are secondary plant metabolites produced in response to damage by pests and other stressful challenges. Among Rutaceae species, FCs are characteristic compounds of citrus peel. FCs are found in citrus fruits together with polymethoxyflavones (PMFs) and coumarins (Cs); the latter are also contained in several spices, particularly cinnamon. These chemicals have been extensively investigated for their wide range of biological activities, making them interesting nutraceuticals for dietary supplements. Arigò et al. [2], in the study “Dietary Intake of Coumarins and Furocoumarins through Citrus Beverages: A Detailed Estimation by an HPLC-MS/MS Method Combined with the Linear Retention Index System”, quantified 35 oxygen heterocyclic compounds (FCs, PMFs, and Cs) in various drinks to obtain informative data useful for the regulatory authorities for the establishment of official limits.
(3) Terpenes are naturally occurring compounds produced by plants that are of great commercial interest in the food, agricultural, cosmetic, and pharmaceutical industries due to their broad spectra of antibacterial, antifungal, anthelmintic, membrane permeation enhancement, and antioxidant biological activities. However, their applications are often limited by their volatility and the requirement for surfactants to produce stable and soluble products. Soto et al. [3], in the study “Yeast Particle Encapsulation of Scaffolded Terpene Compounds for Controlled Terpene Release”, reported the development of a second generation of yeast particle-encapsulated terpene technology that incorporates the stimuli-responsive control of terpene release. Hence the use of biodegradable pro-terpene compounds enabled higher encapsulation stability whilst retaining the full biological activity of the parent terpene compound.
(4) Lemon essential oil (LEO) is a well-known flavoring agent with versatile biological activities. The main components of LEO are represented by monoterpenoids, being a complex mixture of limonene, γ-terpinene, citral, linalool, β-caryophyllene, α-pinene, and β-pinene, exhibiting anti-inflammatory and antioxidant effects. Pucci et al. [4], in the study “Biological Properties of a Citral-Enriched Fraction of Citrus *limon* Essential Oil”, isolated and characterized four citral-enriched fractions of winter LEO. Overall, the reported results encourage the application of EO fractions, enriched in citral, in the nutraceutical industry, not only for their organoleptic properties but also for their protective action against inflammation and oxidative stress.
(5) Although essential oils have a long history of use in medicinal plant, fragrance, and flavour applications, their potential human application is only now being explored. For example, Mastiha essential oil, rich in myrcene, α-pinene, and β-pinene, is obtained from water distillation of the resin of *Pistacia lentiscus* and has a commercial value not only as an aromatic resin but also as a medicinal plant [5]. Papada et al. [6] in the study “An Absorption and Plasma Kinetics Study of Monoterpenes Present in Mastiha Oil in Humans” identify and quantify these bioactive monoterpenes in the plasma following the acute consumption of this Mediterranean essential oil. This is the first study showing the bioavailability of this oil in the plasma as soon as 30 min after consumption.
(6) Conventional pesticides present several problems. An increasing global population has increased the use of pesticides in agriculture [7]. This overuse has led to serious health and ecological issues. Also, the problem is compounded by increasing insecticide and fungicide resistance. There is a movement to use more ‘nature-friendly’ bioactive compounds present in natural products. Plant-derived essential oils with insecticidal, fungicidal, and bacteriocidal activity fall into this category. Certain essential oils can enhance the action of commercially available pesticides. Dassanayake et al. [8] have completed a comprehensive review entitled “Synergistic Field Crop Pest Management Properties of Plant-Derived Essential Oils in Combination with Synthetic Pesticides and Bioactive Molecules: A Review”. These authors review: the historical background and development of natural products in agriculture; the sources and chemical composition of plant-derived essential oils; pesticidal and fungicidal action mechanisms; synergistic and hybridized insect pest management products; synergistic and hybridized fungicidal activity; and novel developments.
(7) Lemon oils are one of the most prominent essential oils traded and consumed today. Thus far, knowledge of a more obscure source, *Backhousia citriodora* (BC), has been limited. Southwell [9] presents a timely and comprehensive review entitled “Backhousia citriodora F. Muell. (Lemon Myrtle), an Unrivalled Source of Citral.” In this allegedly unrivalled source of lemon constituents, details of the taxonomy, etymology, habit, distribution, chemotypes, agronomy, uses, essential oil, oil chemistry, plant bioactivity, toxicology, standards, and commerce are examined. BC is compared with other sources of lemon constituents to determine its superiority. The plant’s applications for flavour, fragrance, and health care are highlighted. The work also studies the plant’s chemistry and its use for determining authenticity via Australian and International standardisation [10,11]. Health-care investigations on both the oil and extract are outlined, and its safety and toxicology (e.g. GRAS status) are highlighted. It also attempts to summarise the current commercial situation regarding production, value, and markets [12].
